# Differential inhibitory response to telcagepant on αCGRP induced vasorelaxation and intracellular Ca^2+^ levels in the perfused and non-perfused isolated rat middle cerebral artery

**DOI:** 10.1186/s10194-017-0768-4

**Published:** 2017-05-30

**Authors:** André Erdling, Majid Sheykhzade, Lars Edvinsson

**Affiliations:** 1 Department of Clinical Sciences, Division of Experimental Vascular Research, Lund University, BMC A13, 221 84 Lund, Sweden; 2 Department of Drug Design and Pharmacology, Faculty of Health and Medical Sciences, University of Copenhagen, Copenhagen, Denmark; 3 Department of Clinical Sciences, Division of Experimental Vascular Research, Lund University, Lund, Sweden

**Keywords:** CGRP, Calcitonin gene related peptide, Telcagepant, Pressurized arteriography, FURA-2, Middle cerebral artery

## Abstract

**Background:**

Calcitonin gene-related peptide (CGRP) is one of the most potent endogenous vasodilators identified to date. The present study elucidates the differential interaction of CGRP, its receptor and the effect of the CGRP-receptor antagonist telcagepant on intracellular Ca^2+^ -levels and tension in rat middle cerebral arteries (MCA) by pressurized arteriography, FURA-2/wire myography and immunohistochemistry.

**Methods:**

A pressurized arteriograph system was used to evaluate changes in MCA tension when subjected to CGRP and/or telcagepant. Intracellular calcium levels were evaluated using a FURA-2/wire myograph system. Localization of the CGRP-receptor components was verified using immunohistochemistry.

**Results:**

Abluminal but not luminal αCGRP (10^-12^-10^-6^ M) caused concentration-dependent vasorelaxation in rat MCA. Luminal telcagepant (10^-6^ M) failed to inhibit this relaxation, while abluminal telcagepant inhibited the relaxation (10^-6^ M). Using the FURA-2 method in combination with wire myography we observed that αCGRP reduced intracellular calcium levels and in parallel the vascular tone. Telcagepant (10^-6^ M) inhibited both vasorelaxation and drop in intracellular calcium levels. Both functional components of the CGRP receptor, CLR (calcitonin receptor-like receptor) and RAMP1 (receptor activity modifying peptide 1) were found in the smooth muscle cells but not in the endothelial cells of the cerebral vasculature.

**Conclusions:**

This study thus demonstrates the relaxant effect of αCGRP on rat MCA. The vasorelaxation is associated with a simultaneous decrease in intracellular calcium levels. Telcagepant reduced relaxation and thwarted the reduction in intracellular calcium levels localized in the vascular smooth muscle cells. In addition, telcagepant may act as a non-competitive antagonist at concentrations greater than 10^-8^ M.

## Background

Calcitonin gene-related peptide (CGRP) is a 37-amino acid neuropeptide belonging to the calcitonin family of peptides, which also includes calcitonin, amylin and adrenomedullin. The peptide, which was first identified in 1982 [1], exists in two forms, αCGRP and βCGRP of which αCGRP is more abundant in the circulation and in the nervous system [2]. CGRP is widely distributed throughout the central, peripheral and enteric nervous systems [3–5] and has strong vasodilatory properties, especially in the microvasculature and in cerebral arteries [6–8]. CGRP is commonly co-localized with substance P in sensory neurons [9]. Cerebral αCGRP is stored in perivascular sensory C-fibers that originates in the trigeminal ganglion. The vasomotor actions of αCGRP are implied in a variety of both physiological and pathological processes, including migraine and other primary headaches as well as subarachnoidal hemorrhage (SAH) [10, 11].

The CGRP receptor is a complex consisting of the G-protein coupled calcitonin receptor-like receptor (CLR) and a single transmembrane receptor activity modifying protein (RAMP1) [12]. CLR may form complexes with other RAMPs with receptor affinity modulation as a result. CLR/RAMP2 has, for instance, a high affinity for adrenomedullin (AM) and constitutes the AM1 receptor [13] while CLR/RAMP3 forms the AM2 receptor with reported affinity for both CGRP and adrenomedullin [14].

There is also evidence of a third, intracellularly located, receptor component protein (RCP), which is thought to stabilize the interaction between the CLR/RAMP1-complex and the G-proteins [15, 16]. In most tissues, receptor activation causes interaction with G_s_ which activates adenylate cyclase (AC) and stimulates the generation of cyclic adenosine monophosphate (cAMP) with subsequent activation of protein kinase A (PKA) leading to opening of K^+^_ATP_- [17] and K_Ca_^2+^ channels [18] and hyperpolarization of smooth muscle cell membrane. A recent study demonstrates that a major portion of CGRP induced vasodilation in MCAs is attributed to activation of Kv7.4 channels [19]. In some vascular beds, for instance in the rat aorta [6], CGRP-induced vasodilation is to some extent mediated via release of nitric oxide from the endothelium rather than only a direct effect on the smooth muscle cells (SMC). The dilatory effect on feline cerebral arteries is however clearly endothelium independent and is associated with activation of AC [20]. The same is true for human cerebral, meningeal and temporal arteries [21–23].

In the present study, we have evaluated in more detail the functional effects of high concentrations of the CGRP-receptor antagonist telcagepant (MK-0974) on αCGRP-induced vasodilatation in isolated middle cerebral arteries (MCA) of rat, using (i) pressurized myography and (ii) FURA-2-AM for measurement of intracellular calcium performed simultaneously with recordings of isometric tension development. We also evaluated the distribution of the CGRP receptor components using immunohistochemistry. The results suggest (i) presence of CGRP receptors on the smooth muscle cells of the MCA, (ii) absence of CGRP receptors on the vascular endothelium, (iii) coupling of receptor-ligand interaction to tension and intracellular calcium and (iv) concentration-dependent antagonism of CGRP-induced vasodilation by telcagepant.

## Methods

The experimental protocol was reviewed and approved by the Animal Protocol Review committee at Lund University, Sweden (M111-04).

Male Sprague-Dawley rats (250 – 350 g; *n* = 20) were anaesthetized with CO_2_ and sacrificed by decapitation. The brain was immediately removed, and placed in oxygenated (95% O_2_ and 5% CO_2_), cold (~ 4° C) buffer solution of the following composition: 119 mM NaCl, 15 mM NaHCO_3_, 4.6 mM KCl, 1.2 mM MgCl_2_, 1.2 mM NaH_2_PO_4_, 1.5 mM CaCl_2_ and 5.5 mM glucose.

The middle cerebral artery (MCA) was carefully isolated from the brain under a stereomicroscope. Each MCA was cut into smaller segments, beginning at the circle of Willis and extending 6-8 mm distally. The dissection was done in cold and oxygenated buffer solution (see composition above).

### Pressurized arteriography experiments

4–6 mm long segments of the MCA were mounted between glass micropipettes and fitted in a pressurized arteriography system (Living Systems, Burlington, VT, USA) as previously described [24]. The artery segments were immersed in buffer solution which was kept at 37° C and continuously aerated with a gas mixture containing 95% O_2_ and 5% CO_2_ with a pH of 7.4. Transmural pressure and luminal perfusion was adjusted to 85 mmHg and 100 μL/min (range 70-100 μL), respectively, by adjusting two fluid reservoirs, which were connected to the afferent (inlet) and efferent (outlet) micropipettes and placed at the appropriate height above the perfused artery segment.

The setup was visualized by a microscope (at 600-fold magnification) connected to a digital camera (Axis, Lund, Sweden). Images recorded by the camera were exported to and stored on a computer. Outer vessel diameter was measured every second and evaluated in the computer software Mary® (Nihil KB, Lund, Sweden).

Following mounting, the perfused vessels were allowed to equilibrate and attain a stable tone. The mean diameter of the vessel segments immediately after mounting was 197.7 ± 4.6 μm and the mean diameter after gaining tone was 132.4 ± 2.0 μm which equals contraction by 33.0 ± 2.0%. Failure to contract at least 10% within one hour disqualified the vessel segment from further experiments. The remaining segments had ATP (10^-5^ M) added to their luminal perfusate in order to evaluate endothelial viability [25]. A dilatory response of at least 10% was considered indicative of a functional endothelium and a structurally intact blood-brain barrier [24, 26]. The mean diameter of the vessel segments increased by 36.1 ± 4.8% upon ATP exposure. The vessel segments were once again allowed to equilibrate (i.e. attain stable tone) before any further experiments were conducted.

Subsequently, αCGRP was added in increasing concentrations (10^-12^ to 10^-6^ M) to either the luminal or the abluminal perfusate in order to evaluate the vascular response.

In a separate experiment, the CGRP antagonist telcagepant was added to either the luminal (10^-6^ M) or the abluminal perfusate (10^-7^-10^-6^ M) 30 minutes before administration of increasing concentrations of abluminal αCGRP.

At the end of each experiment, the vessels were treated with calcium free buffer solution in order to elicit maximal dilatation. The diameter increased by 30.7 ± 4.4% after treatment with calcium free buffer.

### Intracellular calcium measurements

Intracellular calcium measurements were conducted in the dark on middle cerebral artery segments mounted in a wire myograph placed on the stage of an inverted microscope (Leica DMIRBE, Germany). The arteries were loaded with the fluorescent [Ca^2+^]_i_ indicator dye (FURA-2/AM) by incubation in an oxygenated buffer solution (10 μM FURA-2/AM, 0.2% (VV^-1^), anhydrous dimethylsulphoxide (DMSO), 0.01% (VV^-1^), pluronic F-127 and 0.03% (VV^-1^) cremophore EL) for 45 minutes at 37° C. Cremophore EL and pluronic F-127 (dispersing agents or non-ionic detergents) improved the loading with FURA-2/AM by promoting dye dispersion and by preventing FURA-2/AM from precipitation [27]. The loading procedure was performed twice before the vessels were washed and equilibrated for 15 minutes in buffer solution. This also allowed the FURA-2/AM to be converted to active FURA-2 by intracellular esterases.

Determination of intracellular calcium levels was performed by illuminating the vessel segments with light at 340 and 380 nm, respectively. A xenon arc lamp provided high intensity continuous broadband illumination. The emitted light was passed through filters (500 –530 nm) and detected by a photomultiplier (PTI: Photon Technology International, Germany). During the experiments, in which the vessels were precontracted with U44619 (10^-7^ M) and then subjected to incremental concentrations of αCGRP ± telcagepant, fluorescence signals and force signals were continuously captured and processed by a computer (FeliX32 program, Photon Technology International, Monmouth Junction, NJ, U.S.A.). The actual calcium concentrations were calculated according to the equation; [Ca^2+^]_i_ = K_d_×β×[(R-R_min_)/(R_max_-R)], where K_d_ is the dissociation constant of the FURA-2-Ca^2+^ complex (224 nM at 37° C [28]), R is the measured background-corrected ratio between emission at 340 nm excitation and emission at 380 nm excitation, R_max_ and R_min_ are background-corrected ratios under Ca^2+^-saturating and Ca^2+^-free conditions, respectively. The β is the ratio of emission signals when excited at 380 nm during determination of R_min_ and R_max_, respectively. R_min_ and R_max_ were determined in each vessel at the end of the experiment by adding 40 μM ionomycin in calcium free buffer solution and by using buffer-solution containing 5 mM Ca^2+^, respectively [29]. Before calculating the ratio (R) between emission after 340 nm illumination and emission after 380 nm illumination, background fluorescence signals were obtained by quenching the calcium-sensitive FURA-2 fluorescence with 20 mM Mn^2+^ at the end of each experiment [27, 29]. The mean values (*n* = 15) of R_min_, R_max_ and β were 1.12 ± 0.02, 4.59 ± 0.19 and 2.11 ± 0.18, respectively. The values in Ca^2+^-free buffer and the plateau phases were designated to be 0 and 100% for both the [Ca^2+^]_i_ and tension, respectively.

### Immunohistochemistry

MCAs from male Sprague-Dawley rats were carefully dissected from the brain as described above, fixed in 4% paraformaldehyde in phosphate buffer for 1 h, followed by rinsing in Soerensen’s phosphate buffer with increasing concentrations of sucrose. The fixed specimens were then placed into Tissue TEK (Gibo, Invitrogen A/S, Taastrup, Denmark), frozen on dry ice and sectioned into 10-μm-thick slices, which were rehydrated in phosphate buffer solution (PBS) containing 0.25% Triton X-100 for 15 minutes before incubation overnight at +4° C with the following primary antibodies: rabbit polyclonal to RAMP1 diluted 1:200 and sheep polyclonal to CLR (details for the antibodies are summarized in Table 1). All primary antibodies were diluted in PBS containing 0.25% Triton X-100. Sections were subsequently incubated for 1 hour at room temperature with secondary FITC-conjugated donkey anti-rabbit or Dy594-conjugated donkey anti-rabbit antibodies diluted 1:200 in PBS containing 0.25% Triton X. The sections were subsequently washed with PBS and mounted with Vectashield mounting medium (Vector laboratories, Inc. Burlingame, CA, USA). Immunoreactivity was visualized and photographed using a light- and epifluorescence microscope (Nikon 80i; Tokyo, Japan) at the appropriate wavelength. The same procedure was used for the negative controls except that the primary antibodies were omitted resulting in no staining in the tissue except for auto-fluorescence in the internal elastic lamina.

**Table 1:** Details of antibodies used for immunohistochemistry

Primary antibodies, Immunohistochemistry				
Product ID	Detects	Host	Dilution	Supplier
RAMP1 3158	C-terminal of rat RAMP1	Rabbit	1:200	Merck & Co. Inc, USA
CLR 132	C-terminal of rat CRLR	Sheep	1:100	Merck & Co. Inc, USA
Secondary antibodies, Immunohistochemistry				
Product ID	Conjugate and host		Dilution	Supplier
10006588	FITC, goat anti-rabbit		1:100	Cayman chemical, Ann Arbor, MI, USA
713-516-147	Dy594, donkey anti-sheep		1:200	Jackson ImmunoResearch, Europe Ltd., Suffolk, UK

### Drugs

Human α-calcitonin gene-related peptide (αCGRP) (Sigma-Aldrich, Germany) was dissolved in 0.1% bovine serum albumin (BSA) to form a stock solution of 10^-4^ M. 9,11-dideoxy-9α,11α-methanoepoxy PGF_2α_ (U46619, Sigma-Aldrich, Germany), a thromboxane A_2_ agonist, was delivered in methyl acetate (10 mg/ml). A stock solution of 10^-3^ M was prepared by diluting the substance in sterile water. Telcagepant (MK-0974) was synthesized and supplied by the Medicinal Chemistry Department (Merck, West Point, PA, USA) and dissolved in dimethylsulphoxide (DMSO, Sigma-Aldrich, Germany) at a concentration of 10^-2^ M. All stock solutions were stored at -20° C until utilized.

### Data analysis

Relaxations are expressed as percentage of the pre-tension or [Ca^2+^]_i_ level induced by 100 nM U46619. All concentration-response curves were analyzed by iterative non-linear regression analysis using GraphPad Prism 6.03 (GraphPad Corp, San Diego, CA, USA). Each regression line was fitted to a sigmoid equation: E/E_max_ = A[M]^nH^/(A[M]^nH^ + EC_50_[M]^nH^), where E_max_ is the maximal response developed to the agonist, A [M] is the concentration of agonist and n_H_ is a curve-fitting parameter, the Hill coefficient [30]. Sensitivity to agonists is expressed as pD_2_ value, where pD_2_ = -log (EC_50_ [M]), and EC_50_ [M] is the molar concentration of agonist required to produce half-maximal response. Data are expressed as mean ± SEM (n = number of rats) unless otherwise indicated. For experiments performed on the pressurized arteriograph, changes in measured diameters of the vessel segments are expressed as a percentage of the resting diameter. All concentrations expressed indicate the final concentration in the luminal or abluminal compartments of the pressurized arteriograph.

The Kruskal-Wallis test or one-way ANOVA (with Dunn’s or Dunnett’s multiple comparison test) was used when determining differences in E_max_ and pD_2_ between treatment groups. *P*-values < 0.05 were considered significant. NS = not significant.

## Results

### Pressurized arteriography

Telcagepant, luminal or abluminal, did not alter the baseline tone in the pressurized MCAs, indicating no CGRP tone in resting MCAs.

**Fig. 1:**
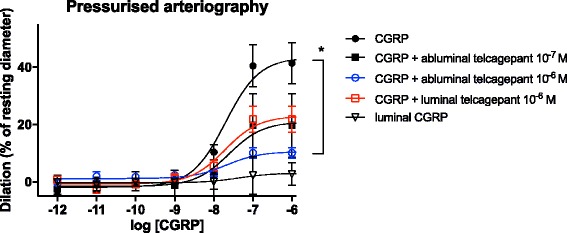
Pressurized arteriography experiment demonstrating the vasodilatory properties of abluminal CGRP on rat MCA and the concentration-dependent inhibition of this relaxation by telcagepant. Values given are means ± SEM, *n* = 4-12. * = *p* < 0,05 for CGRP vs CGRP + telcagepant 10^-6^ M. Lum = luminal application of telcagepant

The administration of αCGRP caused concentration-dependent relaxation of the MCA when given abluminally (E_max_ 41.4 ± 7.4%, pD_2_ 7.73 ± 0.24, *n* = 12). Luminal administration of αCGRP failed to elicit any response at concentrations up to 10^-6^ M (E_max_ 3.1 ± 1.3%, pD_2_ = 7.37 ± 0.65, *n*= 9). Luminal telcagepant (10^-6^M) failed to inhibit the vasodilatory properties of abluminally applied αCGRP (E_max_ 21.85 ± 4.59%, pD_2_ 7.75 ± 0.27, *n* = 5, NS for E_max_). Abluminal telcagepant reduced the dilatory response to abluminal αCGRP significantly when given at high concentration (10^-6^ M) (E_max_ 10.10 ± 1.84%, pD_2_ 7.72 ± 0.30, *n* = 4, *p* = 0.014 for E_max_) while lower concentration of telcagepant (10^-7^ M) failed to do so (E_max_ 19.74 ± 11.06%, pD_2_ 7.65 ± 0.69, n = 4, NS for E_max_) (Fig. 1).

### Intracellular calcium measurements

**Fig. 2:**
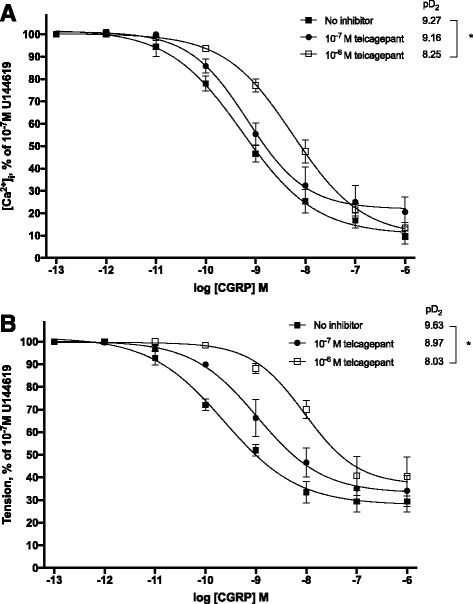
Effect of 0,1μM and 1μM telcagepant on CGRP-induced reduction in [Ca^2+^]_i_ (**a**) and tension (**b**) of rat MCAs pre-contracted with 0,1 μM U46619. Relative tension and [Ca^2+^]_i_ are given as percentages of the initial steady-state levels induced by 0,1 μM U46619. Points represent mean values of n = 3 – 6 separate experiments and vertical bars indicate ± SEM

Incremental administration of αCGRP caused vasodilatation and reduction of intracellular calcium levels. The addition of 10^-6^ M telcagepant significantly offset both the dilatory response and the reduction in intracellular calcium mediated by CGRP (pD_2_ tension 9.63 ± 0.14 vs 8.03 ± 0.18, *n* = 5-6, *p* = 0.02; pD_2_ calcium 9.27 ± 0.11 vs 8.25 ± 0.09, *n* = 4, *p* = 0.03). In contrast, telcagepant at the concentration of 10^-7^ M had no significant inhibitory effect on αCGRP-induced responses (pD_2_ tension 9.63 ± 0.14 vs 8.97 ± 0.20, *n* = 5-6, NS; pD_2_ calcium 9.27 ± 0.11 vs 9.16 ± 0.14, *n* = 3-4, NS) (Fig. 2).

### Immunohistochemistry

**Fig. 3:**
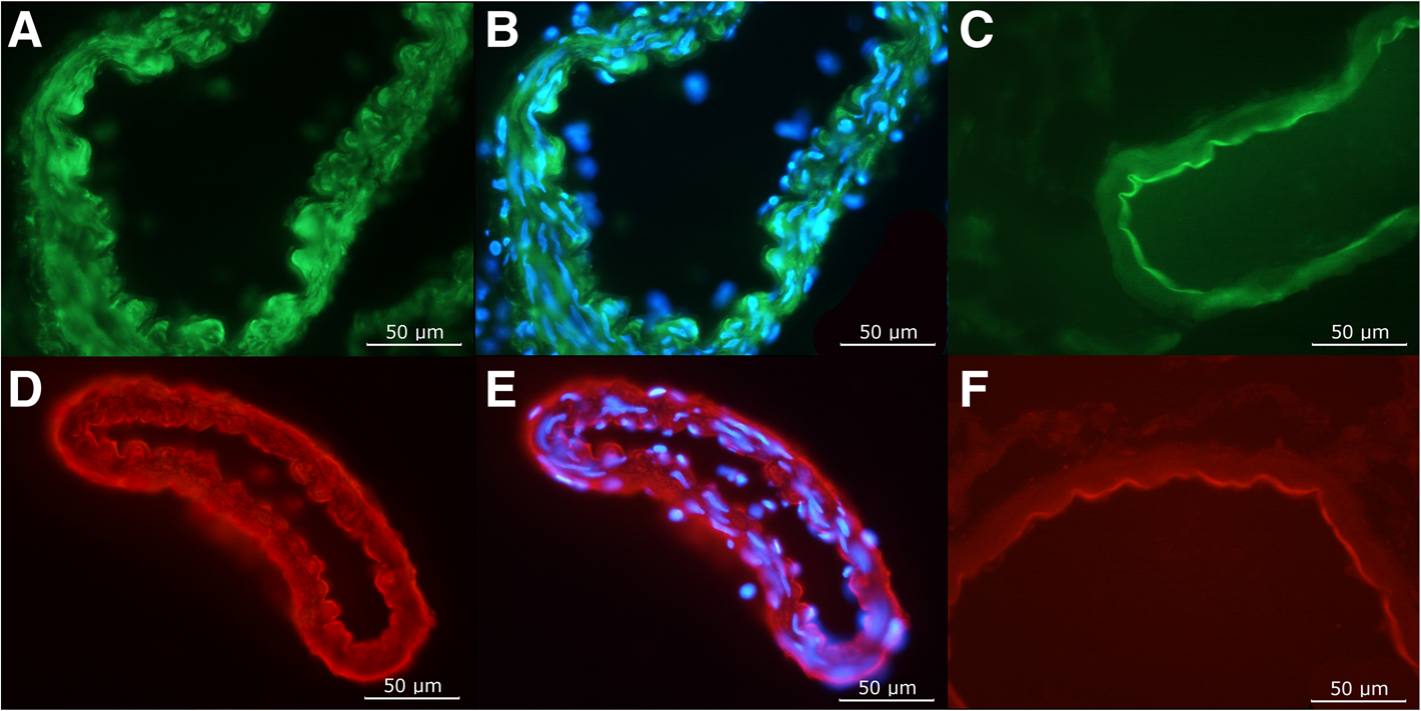
Cross-sections of middle cerebral arteries showing CLR and RAMP1 expression in the smooth muscle cells of the MCA walls. Both receptor antibodies revealed positive staining in the smooth muscle cell layer. A 50 μm marker is inserted. Figure 3a and d demonstrates the expression of CLR and RAMP1 in the smooth muscle cells of the MCA walls. DAPI, a nucleus stain has been added to picture 3b and e. 3c and e are negative controls where the fluorescence staining around the lumen is due to autofluorescence of the lamina elastica interna

MCA segments were first stained with hematoxylin (HTX) and inspected for general morphology; this revealed a thin adventitia, a smooth muscle cell layer, an internal elastic membrane and a single layer of endothelial cells. Both RAMP1 and CLR immunoreactivity could be detected within the smooth muscle cells of the MCA, some RAMP1 was also detected within the endothelial cells but no CLR. Thus, both components of the functional CGRP receptor complex are present in SMC cytoplasm, but not in the endothelial cells (Fig. 3).

## Discussion

The present study has localized the two CGRP receptor elements to the cerebrovascular smooth muscle cells and demonstrates the functional effects of the CGRP-receptor antagonist telcagepant on αCGRP-induced reduction in tension and intracellular calcium concentration in isolated rat MCA segments. We investigated in some detail the dilatory response of αCGRP on precontracted cerebral vessels and found that pre-treatment with telcagepant causes a concentration-dependent inhibition of the dilatory response induced by abluminal αCGRP. This was not seen when telcagepant was administered luminally. Telcagepant did not affect basal tone, hence speaking against CGRP tone in the resting or precontracted MCA. In addition, αCGRP failed to induce dilation when given luminally. This, in combination with the histological localization of the CGRP receptor components in tunica media part of the vasculature, support the opinion that CGRP-receptors are present only in the cerebrovascular smooth muscle cells rather than on the endothelium in the brain vasculature. The finding of weak expression of RAMP1 but no CLR expression could indicate that endothelial cells might express e.g. amylin receptors, but this needs further analysis. The study thus supports once again the important physiological role of CGRP as neuropeptide, namely its presence in perivascular sensory nerve endings and its local release to affect postsynaptic receptors on adjacent vascular smooth muscle cells and subsequent diffusion through the vessel into the blood stream. We also demonstrated a direct correlation between αCGRP-induced dilatory response and alterations in the intracellular calcium levels. Telcagepant was for the first time shown to inhibit the reduction in both tension and in intracellular calcium levels at a given αCGRP concentration. However, some results are a bit contradictory. Telcagepant failed to shift the αCGRP concentration-response curve to the right in the pressurized arteriography experiments. There was, however, a reduction in CGRP-induced maximal vasodilatation (E_max_) when the vessels were subjected to telcagepant, indicating non-competitive/unsurmountable antagonism. In contrast, the wire myography accompanying the measurement of intracellular calcium levels failed to show a drop in E_max_, but could demonstrate a rightward shift of the log [CGRP]-response curve (change in potency) when subjected to telcagepant. The difference between tension development and receptor antagonism in the wire myographs and pressurized arteriography system is interesting. The cause for the discrepancy between results from the two methods could be due to the presence of endothelial CGRP receptors. This scenario seems unlikely since we failed to detect CLR within the endothelium. Difference in vessel diameter could be an issue, but all vessel segments had similar lumen diameter and were taken from the same anatomical location. The advantage of pressurized arteriography lies in its mimicry of actual physiological conditions such as shear stress on the endothelium caused by flow through the vessel lumen and the separation of the luminal and abluminal sides of the vessel wall. It has been demonstrated that vessels in a wire myograph are less sensitive to and respond differently to a variety of agonists (i.e. difference in maximum response and slope of the curve) when compared to vessels in a pressurized system or in vivo [31, 32]. This might be due to the need for precontraction when studying vasorelaxation in the wire myograph, but it may also be due to misinterpretation of actual vasorelaxation as measured in the pressure myograph and reduction of wall tension as measured in the wire myograph model.

The results from the pressurized arteriography are consistent with a recent study where the effect of different concentrations of telcagepant was evaluated on human subcutaneous arteries [33]. Telcagepant seemed to act as a competitive antagonist at relatively low concentrations up to 10^-8^ M but higher concentrations of telcagepant caused a decrease in E_max_ and failed to further shift the agonist log [concentration]-response curve to the right, indicating unsurmountable antagonism. Similar findings were reported when the effect of the CGRP receptor antagonist BIBN4096BS (olcegepant) was tested on human subcutaneous arteries [34]. Further investigation of this phenomenon is needed, especially since the small sample size and the rather large variability might be an issue.

CGRP and its receptor have been under scrutiny for the last decade because of their implied role in migraine and regulation of vasomotor tone [35]. CGRP-containing trigeminal nerves have been shown to surround the major cerebral and cortex pial arteries and stimulation of these nerves releases CGRP which in turn causes direct vasodilation via receptors located on the vascular smooth muscle cells. It has also been suggested that its action on second order neurons may facilitate the transmission of pain to higher cortical areas. There is an ongoing debate regarding whether migraine pain originates in the perivascular nerves, in the CNS or both [10, 36].

Several publications have implied a role for CGRP in the development and prevention of cerebral vasospasm following SAH. Levels of CGRP in cerebrospinal fluid (CSF) and jugular vein blood are increased following SAH [11, 37] as well as during migraine attacks [38].

Telcagepant and olcegepant started as promising agents for the treatment of migraine. Incidence of liver toxicity in chronic studies was, however, the main reason that the projects were eventually terminated just before entry on the market. Despite this, both agents remained as important tools for the understanding of CGRP and its role in cerebrovascular regulation and there are ongoing trials with other small molecules of the “gepant” group, possibly lacking liver toxicity. Since telcagepant is a rather large, hydrophilic substance, this feature might impair its penetration through the blood-brain barrier into the CNS. The neurogenic inflammation induced by substance P and CGRP may, however, aid the translocation of telcagepant into the CNS through increased vascular permeability and reduced integrity of the blood-brain barrier and thus enable interaction with CGRP receptors on the abluminal surface of vessels as well as on perivascular nerves and higher cortical structures.

Low penetration through the blood-brain barrier is one explanation to why high plasma concentrations of telcagepant are needed to treat migraine attacks while vascular effects are seen at lower concentrations in vitro [39]. This is somewhat supported by our study where we compare the effect of CGRP and telcagepant in two different myograph systems. The pressurized arteriography mimics the situation *in vivo* to a greater extent than the wire-myograph setup where telcagepant has direct access to both the luminal and abluminal surfaces of the blood vessel. A previous attempt at comparing the differences in in vivo and in vitro potency of olcegepant and telcagepant [40] reported a large difference in doses needed for in vivo and in vitro effect. Other factors that may explain this discrepancy between clinically effective plasma concentrations and in vitro effects include a high degree of binding to plasma proteins and delayed distribution to the effect compartment.

CGRP acts as an agonist not only at the CGRP receptor, but also on the amylin and adrenomedullin receptors. A functional study on the potency of CGRP, adrenomedullin and amylin at their corresponding receptors report 100-fold lower potency of CGRP at the AM1 and AM2 receptors than at the CGRP receptor [41]. CGRP effects mediated through interaction with the AM receptors cannot be ruled out at the high concentrations used, but the presence of functional AM or amylin receptors on the rat cerebral vasculature seems limited as both of amylin and adrenomedullin only caused weak dilatation when compared to CGRP [42].

## Conclusions

This study demonstrates in detail the relaxant effect of αCGRP on rat MCA. The vasorelaxation is for the first time shown to be associated with a simultaneous decrease in intracellular calcium levels in the cerebral circulation. Telcagepant thwarted the CGRP-induced reduction in tension and intracellular calcium levels of MCAs. When investigated in the pressurized arteriography system, αCGRP produced relaxation only when given abluminally. This observation, together with immunohistochemistry results, strongly suggests an expression of the CGRP receptor complex on the smooth muscle cells in the media rather than on the endothelium. It also implies that CGRP antagonists such as telcagepant have to cross the blood-brain barrier in order to block CGRP induced vasorelaxation in the cerebral circulation. In addition, this study also supports earlier findings suggesting that telcagepant *may* act as an unsurmountable antagonist at concentrations greater than 10^-8^ M. Furthermore, parameters such as wall tension (i.e. transmural pressure), flow, shear stress, myogenic tone, and longitudinal stretch can be confounding factors affecting vascular responses to both agonists and antagonists in vivo. Therefore, our results provide additional evidences for exerting caution when extrapolating the in vitro results to the in vivo situation.

## Abbreviations

AM: Adrenomedullin
BSA: Bovine serum albumin
CGRP: Calcitonin gene-related peptide
CLR: Calcitonin receptor-like receptor
DMSO: Dimethyl sulphoxide
MCA: Middle cerebral artery
NS: Not significant
PKA: Protein kinase A
RAMP1/2/3: Receptor activity modifying peptide 1/2/3
RCP: Receptor component protein
U46619: 9,11-dideoxy-9α,11α-methanoepoxy PGF_2α_, a thromboxane A_2_ agonist
